# Use of Non-*Saccharomyces* Yeast Co-Fermentation with *Saccharomyces cerevisiae* to Improve the Polyphenol and Volatile Aroma Compound Contents in Nanfeng Tangerine Wines

**DOI:** 10.3390/jof8020128

**Published:** 2022-01-27

**Authors:** Ahui Xu, Yiwen Xiao, Zhenyong He, Jiantao Liu, Ya Wang, Boliang Gao, Jun Chang, Du Zhu

**Affiliations:** 1 Key Laboratory of Bioprocess Engineering of Jiangxi Province, College of Life Sciences, Jiangxi Science and Technology Normal University, Nanchang 330013, China; ah2815781702@outlook.com (A.X.); xyw1152858687@163.com (Y.X.); hzy379344599@163.com (Z.H.); jxwangya@126.com (Y.W.); gaoblspinach@aliyun.com (B.G.); changjun@jxstnu.edu.cn (J.C.); 2 Key Laboratory of Protection and Utilization of Subtropic Plant Resources of Jiangxi Province, College of Life Sciences, Jiangxi Normal University, Nanchang 330022, China

**Keywords:** tangerine wine, pure fermentation, sequential fermentation, mixed fermentation, phenolic compounds, volatile flavor compounds

## Abstract

This study attempted to improve the polyphenol and volatile aroma compound contents in Nanfeng tangerine wines using non-*Saccharomyces* yeast and *Saccharomyces cerevisiae*. The effects of fermentation with pure cultures of *Candida ethanolica*, *Hanseniaspora guilliermondii* and *Hanseniaspora thailandica*, as well as in sequential and mixed inoculations (1:1 or 1:100 ratio) with *S. cerevisiae* in Nanfeng tangerine wines were evaluated. *C. ethanolica* was found to produce the most polyphenols (138.78 mg/L) during pure fermentation, while *H. guilliermondii* produced the most volatile aroma compounds (442.34 mg/L). The polyphenol content produced during sequential fermentation with *S. cerevisiae* and *H. guilliermondii* (140.24 mg/L) or *C. ethanolica* (140.21 mg/L) was significantly higher than other co-fermentations. Meanwhile, the volatile aroma compounds were found to be more abundant in *S. cerevisiae*/*H. guilliermondii* mixed fermentation (1:1 ratio) (588.35 mg/L) or *S. cerevisiae*/*H. guilliermondii* sequential fermentation (549.31 mg/L). Thus, *S. cerevisiae*/*H. guilliermondii* sequential fermentation could considerably boost the polyphenol and volatile aroma component contents in Nanfeng tangerine wines. The findings of this study can be used to drive strategies to increase the polyphenol content and sensory quality of tangerine wines and provide a reference for selecting the co-fermentation styles for non-*Saccharomyces* yeast and *S. cerevisiae* in fruit wine fermentation.

## 1. Introduction

Nanfeng tangerine (*Citrus reticulata* Blanco) is a well-known delicacy used in China for its flavor and nutrition with a long cultivation history (about 1300 years) and widespread production [1,2]. Tangerine wines not only retain the flavor of Nanfeng tangerines but are also associated with the production of a variety of flavor components and biologically active compounds during the alcoholic fermentation process [3], including polyphenols [4]. Tangerine wines are produced to reduce surplus inventory and fruit degradation caused by overproduction; this helps increase the shelf life and value of Nanfeng tangerines [3,5,6]. However, currently available tangerine wines are uncompetitive due to their restricted flavor profiles, which are determined by fermentation procedures, fermentation strains and other characteristics [6,7,8,9,10,11].

Spontaneous fermentation is the most traditional method for fermenting fruit wines with complex flavors in wineries, although this method is unpredictable due to the existence of diverse bacteria. To allow regulated operations and consistent product quality, industrial fermentation has quickly replaced spontaneous fermentation in fruit wine processing. Due to its high fermentative activity, *S. cerevisiae* has become the most-utilized strain in commercial fruit wine fermentation [12,13]. However, single fermentation with *S. cerevisiae* has greatly reduced biodiversity throughout the wine-making process, resulting in the production of wines with a consistently inferior taste and fragrance [14].

Several studies published in the last few years have examined the role of non-*Saccharomyces* yeasts in fruit wine fermentation, concluding that non-*Saccharomyces* yeast strains impart high concentrations of aroma compounds and some volatile compounds with distinct aromas as well as low concentrations of volatile acids into fruit wine [5,8,9,10,11]. Several non-*Saccharomyces* yeasts, on the other hand, may produce repugnant volatile metabolites during fermentation, such as high concentrations of ethyl acetate, acetic acid, alcohols, volatile phenols and aldehyde [6,15], whereas others cannot tolerate harsh fermentation conditions such as biological competition, high concentrations of ethanol and SO_2_, a low pH [12,16,17]. It has been demonstrated that using non-*Saccharomyces* yeast in mixed cultures with *S. cerevisiae* strains can be used to increase the smell, flavor and complexity of fruit wines [6,7,10,14,18,19]. Two forms of co-fermentation have been demonstrated: mixed fermentation and sequential fermentation. The first approach involves the concurrent inoculation of *S. cerevisiae* and non-*Saccharomyces* yeasts, whereas the second method requires first inoculating non-*Saccharomyces* yeasts and then adding *S. cerevisiae* to the fermentation system [8]. While co-fermentation of distinct strains produces complex metabolites [8,14], the interaction between the strains may also limit their development and have a harmful influence on fruit wine fermentation [11,20]. The selection of suitable strains and fermentation conditions remains an open question.

Wine quality can be determined not just by the sensory quality but also by the presence of bioactive metabolites, such as polyphenols [21]. Polyphenols are secondary metabolites produced by plants via the shikimic acid pathway. They can improve the color, astringency, bitterness, body, mouthfeel, fullness, complexity and structure of fruit wines [21]. Tangerines contain abundant amounts of polyphenols, mainly flavonoids and phenolic acids [22]. Fermentation can improve the yield and change the structure of polyphenols, possibly due to the microbial enzymes that degrade the cell wall structure and promote the release of binding phenolic compounds in plant cell walls as well as transforming phenolic compounds into other metabolites [4].

*C. ethanolica* (Crabtree positive) [23], *H. guilliermondii* (Crabtree positive) [24] and *H. thailandica* (Crabtree negative) [25], three wild non-*Saccharomyces* yeast strains, were recovered from spontaneously fermented tangerine wines in our laboratory. The high amounts of esters and volatile acids by these non-*Saccharomyces* yeasts during natural fermentation increased the smell and flavor qualities of Nanfeng tangerine wines [3]. In this study, these non-*Saccharomyces* yeasts were used to improve the quality of tangerine wines. The effects of pure fermentation and sequential fermentation with *S. cerevisiae* as well as 1:1 and 1:100 mixed fermentation on polyphenols, volatile aroma compounds, ethanol and organic acids in tangerine wines were detected in this study. The main aim was to assess the effects of *C. ethanolica*, *H. guilliermondii* and *H. thailandica* on the polyphenols and volatile aroma compound contents in Nanfeng tangerine wines when used in mixed fermentations with *S. cerevisiae*.

## 2. Materials and Methods

### 2.1. Tangerine Juice Preparation

Nanfeng tangerines were picked from Nanfeng County, Jiangxi Province, in 2020.

Tangerine juice (pH 4.16, 14.6 Brix total soluble solids, 130.14 g/L initial sugar, 8.96 g/L total acidity expressed as citric acid) was made by peeling, crushing, and deslagging fresh tangerine. The SO_2_ and total sugar contents in the tangerine juice were adjusted to 50 mg/L [26] and 200 g/L with K2S2O5 and sucrose, respectively.

An aliquot of 800 mL tangerine juice was immediately transferred to a 1000 mL sterile Duran container. Following that, it was pasteurized for 7 min at 95 °C and chilled to room temperature in an ice bath [8]. Each bottle lid was cushioned with a rubber gasket and 2 2-way valves fitted with 0.22 μm nylon membrane filters. One valve was fitted with a rubber tube and submerged in the juice to facilitate syringe sampling, while the other valve was put above the juice level to allow carbon dioxide to be released from the headspace.

### 2.2. Yeast Strains and Culture Media

*C. ethanolica* (Ce), *H. guilliermondii* (Hg) and *H. thailandica* (Ht) were identified and maintained in our laboratory after spontaneous tangerine wine processes. *S. cerevisiae* (Angel yeast) was obtained from Angel Yeast Co., Ltd. (Yichang, Hubei, China) and was stored at 80 °C before usage in our laboratory.

The inocula were prepared using Yeast Extract Peptone Dextrose Medium (YPD). The Wallerstein laboratory nutrition agar (WL) (Haibo, Qingdao, China) medium enables the potential identification of yeast cells based on the colony’s color and form [27]. Lysine (LYS) agar (Haibo, Qingdao, China) is a selective medium that is incompatible with *S. cerevisiae* growth and is used to count non-*Saccharomyces* yeast strain cells during co-fermentation [7].

### 2.3. Fermentation Process

*S. cerevisiae* and 3 strains of non-*Saccharomyces* yeast were cultured for 24 h at 28 °C with shaking at 160 rpm (Shanghai Yiheng Scientific Instrument Co., LTD., THZ-300, Shanghai, China) twice in succession in sterile YPD media. Following that, centrifugation for 5 min at 4000 rpm (Shanghai Luxiangyi Centrifuge Instrument Co., LTD., TG16-WS, Shanghai, China) was employed to extract yeast cells from broths prior to using them for tangerine juice fermentation.

For pure fermentation, 107 CFU/mL of a single yeast strain was added to tangerine juice. Sequential fermentation meant that *S. cerevisiae* was inoculated into tangerine juice 48 h later than non-*Saccharomyces* yeast. At that point, the residual sugar content was 115 g/L. Three different combinations (Ce-10-Sc, Hg-10-Sc and Ht-10-Sc) were used, with an inoculum ratio of 1:1 and a cell quantity of 107 CFU/mL, respectively. In mixed fermentations, *S. cerevisiae* was inoculated concurrently with non-*Saccharomyces* yeast species at the same cell concentration of 107 CFU/mL (Sc:Ce-1:1, Sc:Hg-1:1, Sc:Ht-1:1). To minimize *S. cerevisiae* competition and the effect of high ethanol concentrations, as well as to reduce the workload in comparison with that of sequential fermentation, we performed mixed fermentation with an inoculum ratio of 1:100 105 CFU/mL *S. cerevisiae* plus 107 CFU/mL non-*Saccharomyces* [7]. Three combinations were grouped (Sc:Ce-1:100, Sc:Hg-1:100, Sc:Ht-1:100). The experimental groups are listed in Table 1.

Each experiment was repeated 3 times, and the fermentation temperature was maintained at 28 °C. Daily residual sugar concentrations and yeast cell counts were used to monitor the fermentation process of tangerine wines. Fermentation was judged to be complete when the residual sugar concentrations remained constant. Both the juice and tangerine wines were centrifuged at 4500 rpm for 10 min after fermentation to remove yeast cells and precipitates. The supernatants were stored at a temperature of 80 °C until analysis.

### 2.4. Yeast Counting

Daily samples were gathered throughout the tangerine wine fermentation process, and the yeast cells were evaluated using the sequential dilution technique on WL Agar and LYS Agar. After that, the plates were incubated at 28 °C for two days. The quantity of yeast cells in each sample was measured using the plate counting technique [7]. Each sample was quantified three times.

### 2.5. Physicochemical Analysis

The anthrone-sulfuric acid test of Yu et al. [28] with minor modifications was used to determine the residual sugar content in tangerine wines. Every 1 mL of the sample was mixed with 4 mL anthrone reagent (0.1% anthrone and H_2_SO_4_). The mixture was incubated in boiling water for 10 min and chilled before reading the absorbance at 620 nm. The total acid content (expressed as g/L of citric acid) of the tangerine juice and wine was determined using the sodium hydroxide titration technique by the GB 12456-2021. Alcohol concentrations were determined using gas chromatography (Scion, GC 456C, Shanghai, China) by the GB/T 15038-2006. The pH value of tangerine wines was determined with a pH meter (Shanghai Yi Electrical Scientific Instrument Co., LTD., PHS-3C, Shanghai, China). All experiments were repeated three times.

### 2.6. Flavonoid, Phenolic Acid and Organic Acid Compound Analysis

HPLC (Agilent Technologies, 1260 Infinity, Palo Alto, CA, USA) was used to analyze the flavonoid, phenolic acid and organic acid contents. Analyses were performed in triplicate.

A chromatographic column, Agilent Eclipse XDB-C18 (4.6 mm × 250 mm, 5 μm), was employed to identify flavonoid components in tangerine wines. The gradient elution protocol is described in Table 2, and a flow rate of 1.0 mL/min was used. The column temperature was set to 30 °C, the quantitative wavelengths used were 283 and 330 nm, the scanning range was 200 to 400 nm, and the injection volume was 10 μL. The retention period was qualitative, but the procedure used to determine the external standard was quantitative. At a wavelength of 283 nm, eriocitrin, narirutin, hesperidin, nehesperidin, didymin and hesperetin were examined. At 330 nm, nobiletin was examined.

The phenolic acid content of tangerine wines was determined using a chromatographic column of Ultimate AQ-C18 (4.6 mm × 250 mm, 5 μm). The gradient elution protocol is described in Table 3, and a flow rate of 1.0 mL/min was used. The column was preheated to 40 °C, and a 10 μL sample was injected. The quantitative wavelengths used were 260 and 320 nm, the scanning range was 200–400 nm, the retention duration was qualitative, and the external standard technique used was quantitative. The protocatechuic acid and p-hydroxybenzoic acid peak areas were determined at 260 nm. The peak areas of chlorogenic acid, caffeic acid, p-coumaric acid, ferulic acid and erucic acid were measured at 320 nm.

The organic acid contents in tangerine wines were determined using an Ultimate AQ-C18 column (4.6 mm × 250 mm, 5 μm) [3]. As the mobile phase, 0.025 percent trifluoroacetic acid solution/methanol (95:5, *v*/*v*) was used. The flow rate was set to 0.8 mL/min, and the determination wavelength was set to 210 nm with a 10 μL injection volume. The quantities of oxalic acid, malic acid, vitamin C, acetic acid, citric acid, succinic acid, fumaric acid, maleic acid and lactic acid were determined using calibration curves generated from the examination of external standards at various concentrations.

### 2.7. Electronic Nose

The Gemini electronic nose (Alpha MOS, FOX4000, Toulouse, France) was used to determine the level of similarity between scent profiles after fermentation. The E-nose study was based on prior studies [29,30]. A total of 5 mL of each tangerine wine sample was placed in a 20-milliliter glass vial and sealed with a Teflon rubber lid. The vial containing the tangerine wine sample was left at room temperature for 30 min, during which time the volatiles from the wines were collected in the headspace. The measurement period was 90 s, which provided sufficient time for the sensors to stabilize their signal levels. When the measurement was complete, the electronic nose program recorded the data for later PCA analysis. The electronic nose’s six metal oxide sensors are listed in Table 4. Different sensors are receptive to various volatile chemicals. Each wine sample was quantified three times.

### 2.8. Volatile Aroma Composition Analysis

The volatile aroma components were extracted using a 50/30 μm DVB/CAR/PDMS fiber in a headspace solid-phase microextraction (HS-SPME) (Supelco, Bellefonte PA, SA) and analyzed using an Agilent 7890B gas chromatography system linked to an Agilent 7000C mass spectrometer using an HP-5MS capillary column (30 m × 250 μm × 0.25 μm). The extraction, analysis and identification of volatile aroma compounds were conducted as described by Hu et al. [5]. A total of 5 milliliters of filtered tangerine wine, 5 μL of the internal standard cyclohexanone (10 mg/mL in ethanol) and 1.5 g of NaCl [26,31] were combined in a 20 mL headspace vial sealed with a screwed top and a 1.5 mm thick Teflon septum. After agitating and equilibrating the solution for 20 min at 40 °C, a fiber was placed through the vial septum and exposed to the headspace for 52 min at 40 °C.

The carrier gas, helium, was maintained at a flow rate of 1.2 mL/min. The column temperature program was set as follows: 40 °C for 2 min, 180 °C at a rate of 4 °C/min for 2 min and 250 °C at a rate of 10 °C/min for 5 min. The detector and ion source were tuned at 250 °C and 200 °C, respectively. At a potential of 70 eV, the mass spectrometer was operated in electron impact mode.

We performed semiquantitative using cyclohexanone as an internal standard. The content of volatile aroma compounds were determined by comparing the GC peak areas of the volatile compound to the GC peak areas of the internal standard [5,32]. To obtain odor patterns, the odor activity value (OAV) was computed as the ratio of the concentrations of a flavor ingredient to its odor threshold (OT) [33]. Volatile aroma molecules containing OAV ≥ 1 were determined to be odor-active. The greater the OAV of volatile compounds, the greater their contributions to the wine flavor [34,35].

### 2.9. Statistical Analysis

All data with error bars are expressed as mean ± standard deviation. SPSS 19.0 was used to run statistical analyses. SIMCA 13.0 was used to conduct a principal component analysis (PCA) to determine the most important volatile fragrance components and functional active substances in various fermentations.

## 3. Results and Discussion

### 3.1. Sugar Consumption Kinetics and Growth Kinetics of Yeast Strains during Fermentation

**Figure 1:**
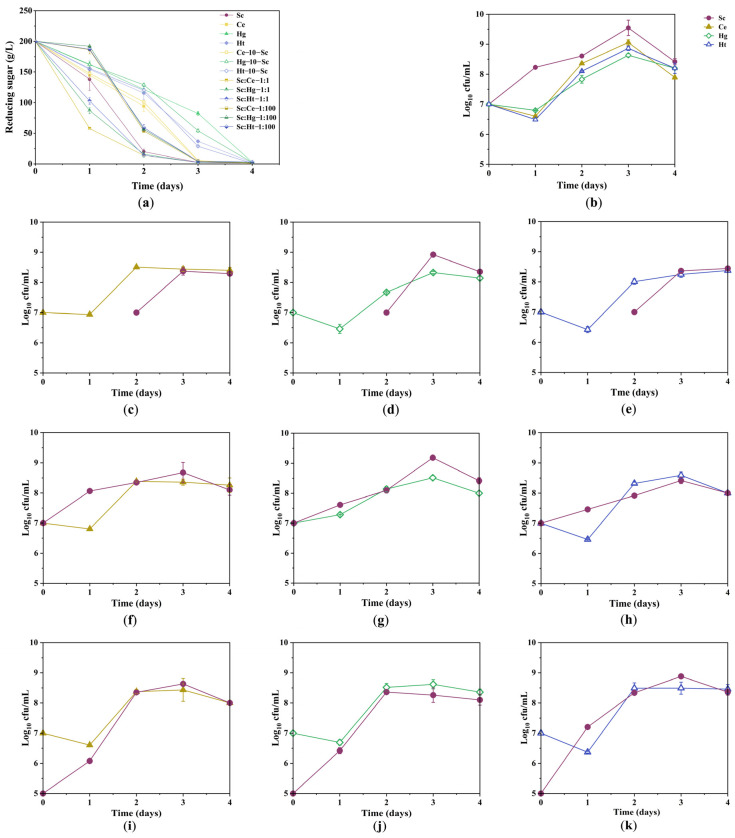
Sugar consumption kinetics and growth kinetics of yeast strains during fermentation. (**a**) Sugar consumption kinetics. (**b**) The growth kinetics of pure fermentations. (**c**–**e**) The growth kinetics of sequential fermentations with *S. cerevisiae*. (**f**–**h**) The growth kinetics of mixed fermentations with *S. cerevisiae* (1:1). (**i**–**k**) The growth kinetics of mixed fermentation with *S. cerevisiae* (1:100). Abbreviations: Sc, *S. cerevisiae*; Ce, *C. ethanolica*; Hg, *H. guilliermondii*; Ht, *H. thailandica*. Data shown are the mean ± SD of triplicate.

To evaluate the effects of pure fermentation, sequential fermentation, 1:1 mixed fermentation and 1:100 mixed fermentation on the growth of yeast, the growth kinetics and sugar consumption kinetics of yeast strains during different fermentation methods were analyzed (Figure 1). Fermentation was completed 3–4 days after inoculation. In pure fermentation, Sc and Ce possessed a stronger fermentation capacity than other yeasts (Figure 1a). These non-*Saccharomyces* yeast strains grew normally within 3–4 days, reaching biomasses of 1.0 × 10^8^–1.0 × 10^9^ CFU/mL (Figure 1b). The maximum biomass produced with pure Sc fermentation was higher than that produced with non-*Saccharomyces* yeast pure fermentation. Additionally, three non-*Saccharomyces* yeasts were quickly inhibited with a dramatic fall in biomass on the first day of fermentation and then continued to develop normally. According to the law of microbial growth, microorganisms entering a new environment will appear to lag in their phase of growth. Additionally, the non-*Saccharomyces* yeasts adapt to the tangerine juice microenvironment more weakly than *S. cerevisiae* [6,36]. Therefore, three non-*Saccharomyces* yeasts’ growth lag phase was extended, and biomass was decreased on the first day of fermentation. Furthermore, the pH value determination results showed that the pH of non-*Saccharomyces* yeast pure fermentations was greater than those of pure Sc fermentation and co-fermentations (Table S1), and the low pH environment may have inhibited the growth of non-*Saccharomyces* yeast, which is consistent with previous reports [6]. In co-fermentation, the fermentation capacity of 1:1 mixed fermentation was stronger than that of other co-fermentations, and the fermentation was completed within 2–3 days after inoculation. The inoculation of these non-*Saccharomyces* yeast strains negligibly affected the maximum biomass of Sc in co-fermentations compared with pure Sc fermentation (Figure 1a–k), indicating that non-*Saccharomyces* yeast and Sc could grow normally during co-fermentation. The suppression of non-*Saccharomyces* yeast strains was most likely a result of Sc’s intense nutritional competition or lethal factors [37,38].

### 3.2. Polyphenols

**Figure 2:**
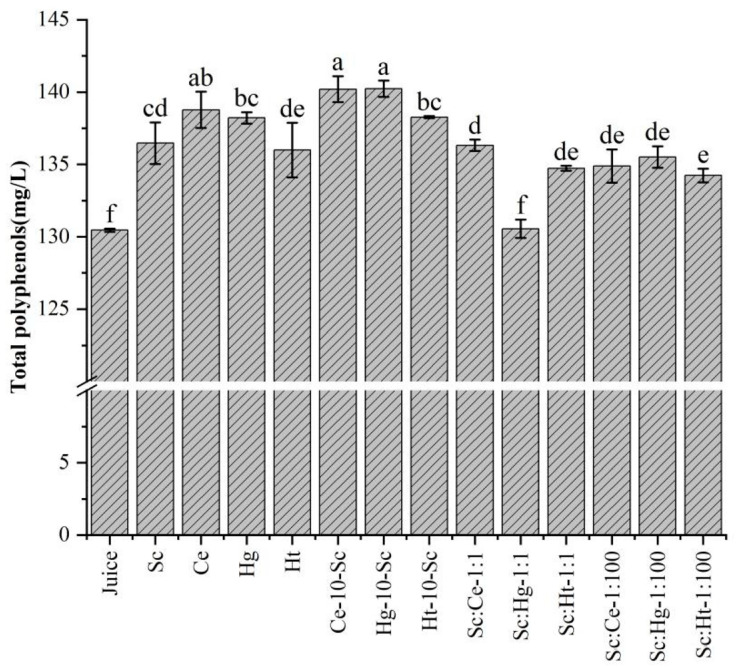
The total polyphenols in tangerine juice and tangerine wines. Abbreviations: Sc, *S. cerevisiae*; Ce, *C. ethanolica*; Hg, *H. guilliermondii*; Ht, *H. thailandica*. Data shown are the mean ± SD of triplicate, values with different Roman letters in the same row indicating significant differences at *p* < 0.05 (Duncan’s test).

Polyphenols are secondary metabolites, mainly flavonoids and phenolic acids, produced in plants through the shikimic acid pathway. To evaluate the effects of pure fermentation, sequential fermentation, 1:1 mixed fermentation and 1:100 mixed fermentation using non-*Saccharomyces* yeast and Sc, the polyphenol content in tangerine wines produced with different fermentation methods was analyzed (Figure 2). Tangerine wines produced through pure Ce or Hg fermentation had the largest quantities of polyphenols. In co-fermentation, sequential fermentation significantly increased the content of polyphenols in tangerine wines compared with other co-fermentation methods. In particular, the contents of polyphenols in tangerine wines produced through Ce-10-Sc or Hg-10-Sc sequential fermentation were the highest among all fermented tangerine wines. These findings indicate that co-fermentation with non-*Saccharomyces* yeast and *S. cerevisiae* can increase the polyphenol content in fruit wines. This is a similar finding to what was reported in a recent study that used non-*Saccharomyces* yeast and lactic acid bacteria in Co-inoculated fermentations with *S. cerevisiae* to improve the phenolic content of Syrah wine [21]. Cellulolytic enzymes, lignin-decomposing enzymes and pectin-decomposing enzymes produced during microbial fermentation can effectively release bound and free polyphenols from the plant substrate by decomposing chemical components from the plant cell wall, leading to an increase in polyphenols in fermented products [4]. Among these enzymes, β-glucosidase has been widely reported during the fermentation of *S. cerevisiae* [4], *H. guilliermondii* [39] and *H. thailandica* [40]. The glycosidases produced by the majority of non-*Saccharomyces* yeasts are more active and tolerant to alcohol than those produced by Sc [9,41]. Furthermore, because Ce is tannin-tolerant and possesses tannase activity [42], tannase may be a possible factor in the release of polyphenols that degrade the cell wall matrix. The higher polyphenol content produced in sequential fermentation may be explained by the fact that delaying the addition of Sc increases the likelihood of non-*Saccharomyces* yeasts producing highly active degradation enzymes, and subsequent addition of other enzymes produced by Sc results in increased enzyme activity and increased content of polyphenols in the fermentation system.

#### 3.2.1. Flavonoids

**Figure 3:**
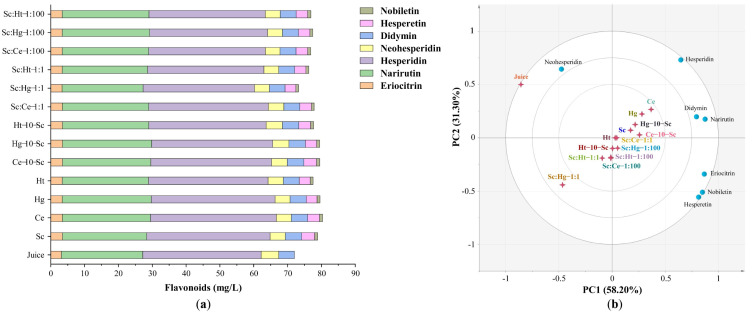
The content of flavonoids in tangerine juice and tangerine wines. (**a**) The content of flavonoids detected by HPLC. (**b**) The principal component analysis of flavonoids. Abbreviations: Sc, *S. cerevisiae*; Ce, *C. ethanolica*; Hg, *H. guilliermondii*; Ht, *H. thailandica*. Data shown are the mean ± SD of triplicate, values with different Roman letters in the same row indicating significant differences at *p* < 0.05 (Duncan’s test).

The flavonoid levels in tangerine wines produced with different fermentation methods are shown in Figure 3a. For quantification purposes, calibration curves were drawn for each chemical compound (Table S2). The total flavonoid content in tangerine wines was significantly higher than in tangerine juice. In pure fermentations, Sc, Ce and Hg significantly increased the content of total flavonoids in tangerine wines. In the co-fermentations, the total flavonoid content in tangerine wines produced by Ce-10-Sc and Hg-10-Sc sequential fermentation was significantly higher than that produced with other co-fermentation styles (Figure 3a). This can probably be ascribed to the enzymes produced by Sc, Ce or Hg being more active in cell wall matrix degradation. Hesperidin and narirutin were the primary flavonoids found in tangerine juice and tangerine wines. Alcoholic fermentation degraded neohesperidin and hesperidin and created hesperetin and nobiletin, contributing to the bitterness reduction in tangerine wines [43]. Hesperetin concentrations in tangerine wines were significantly higher in the Sc and Ce-10-Sc sequential fermentations. Additionally, the tangerine wines produced with Ce-10-Sc sequential fermentation had significantly more nobiletin than those produced with other co-fermentation styles (Table S1). Hesperetin is abundant in fruits, vegetables and traditional Chinese medicinal herbs as glycosides such as hesperidin and neohesperidin. It possesses antioxidant, anti-inflammatory, anti-carcinogenic, anti-allergic and epigenetic modification effects [44,45]. Nobiletin has garnered considerable interest due to its favorable health effects, which include anti-carcinogenic, anti-inflammatory, anti-atherogenic, anti-diabetic and anti-obesity actions [46].

To investigate the potential associations among the various fermentation methods, yeast strains and flavonoid profiles, a principal component analysis with seven flavonoid components and thirteen fermentation groups was used (Figure 3b). The results indicated that both pure and sequential fermentations with Ce and Hg clustered significantly with several flavonoids, including narirutin and didymin, implying that the functional activity of tangerine wines fermented using these methods is related to narirutin and didymin. These findings demonstrate that varied fermentation procedures result in the production of a variety of flavonoids with varying levels of biological activity in tangerine wines.

#### 3.2.2. Phenolic Acids

**Figure 4:**
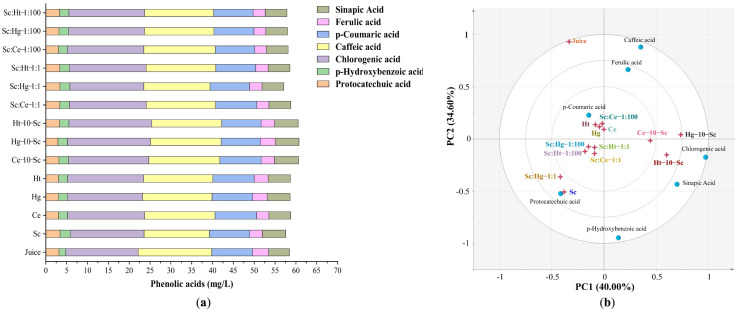
The content of phenolic acids in tangerine juice and tangerine wines. (**a**) The content of phenolic acids detected by HPLC. (**b**) The principal component analysis of phenolic acids. Abbreviations: Sc, *S. cerevisiae*; Ce, *C. ethanolica*; Hg, *H. guilliermondii*; Ht, *H. thailandica*. Data shown are the mean ± SD of triplicate, values with different Roman letters in the same row indicating significant differences at *p* < 0.05 (Duncan’s test).

The level of phenolic acids in tangerine wines produced by different fermentation methods is summarized in Figure 4a. The total phenolic acid concentrations in tangerine wines showed a significant increase in pure fermentation with non-*Saccharomyces* yeast compared with Sc. Co-fermentation resulted in the highest concentration of major phenolic acid components in sequentially fermented tangerine wines, exceeding both other co-fermentation procedures and pure fermentations. The primary phenolic acids present in tangerine juice and tangerine wines were chlorogenic acid and caffeic acid, with the maximum concentrations of chlorogenic acid and caffeic acid being 19.91 and 17.65 mg/L, respectively (Table S1). Caffeic acid and chlorogenic acid can scavenge various free radicals, including superoxide anions and hydroxy radicals [47]. The concentration of chlorogenic acid in tangerine wines was much higher in sequential fermentations than in other fermentation styles. Caffeic acid and ferulic acid were highly unstable during fermentation; their concentrations were decreased in all treatments. In all treatments, the concentrations of p-Hydroxybenzoic acid and chlorogenic acid were significantly elevated. Simultaneously, the protocatechuic acid and p-coumaric acid levels showed irregular changes across all treatments.

According to the PCA results for phenolic acids (Figure 4b), the same fermentation techniques, except juice and Sc pure fermentation, clustered together. The results reveal that fermentation methods have a greater impact on the phenolic acid profiles of tangerine wines than yeast strains. This finding differs from that of a recent study [8], in which yeast species were found to contribute more to changes in the chemical makeup of bilberry wine than the fermentation type. This could be due to the varied yeast strains utilized or the diverse chemical compositions analyzed.

### 3.3. Volatile Aroma Compounds

**Figure 5:**
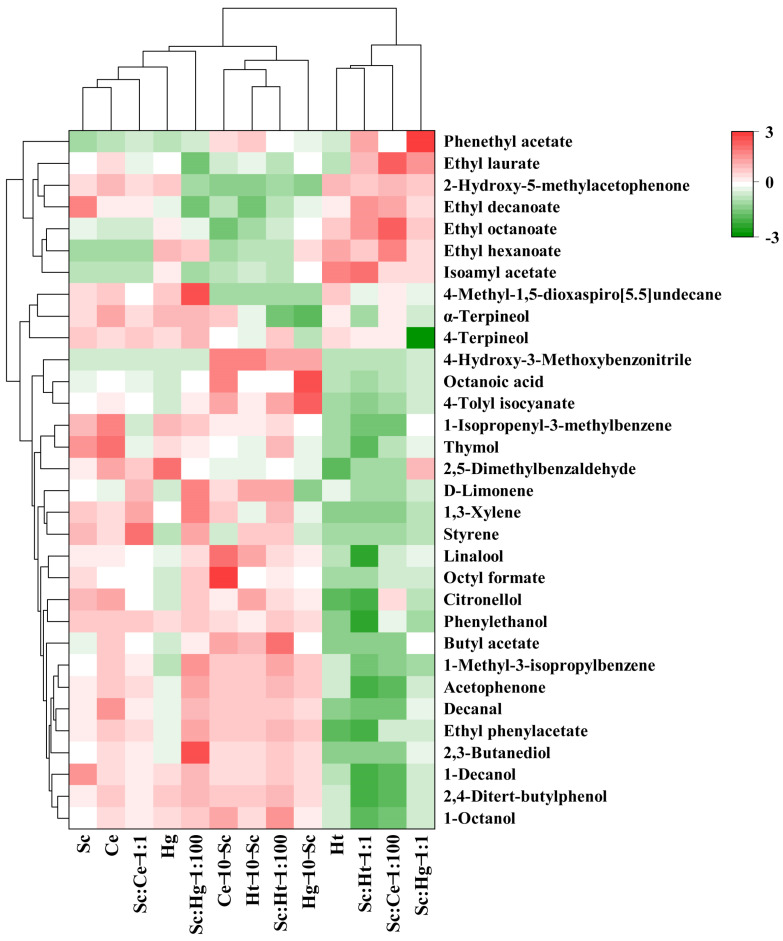
Hierarchical cluster analysis of volatile aroma compounds in tangerine wines. Abbreviations: Sc, *S. cerevisiae*; Ce, *C. ethanolica*; Hg, *H. guilliermondii*; Ht, *H. thailandica*.

As seen in Figure 5, 32 volatile chemicals were found in tangerine wines, including 4 higher alcohols, 10 esters, 7 terpenes, 3 aldehydes and ketones, 2 phenols, 1 acid and 5 others. Table S3 provides the average and standard deviations.

In pure fermentation, compared with Sc, non-*Saccharomyces* yeast strains produced higher contents of volatile aroma compounds. Pure Hg fermentation produced the maximum amount of volatile aroma compounds (442.34 mg/L), followed by pure Ce (382.55 mg/L), Ht (382.03 mg/L) and Sc fermentations (371.41 mg/L). In co-fermentation, the Ce-10-Sc and Sc:Ht-1:100 co-fermentations produced lower concentrations of volatile aroma compounds compared with the pure fermentations, whereas the other co-fermentations were associated with significantly increased concentrations of volatile aroma compounds in tangerine wines. The Sc:Hg-1:1 mixed fermentation generated the maximum amount of volatile aroma compounds in tangerine wines (588.35 mg/L), followed by Hg-10-Sc (549.31 mg/L) and Ht-10-Sc (518.66 mg/L) sequential fermentations (Table S3). These results indicate that co-fermentation of selected non-*Saccharomyces* yeast strains with SC is a promising approach to improve the sensory quality of tangerine wines.

During fermentation, yeasts convert sugar to ethanol, producing a variety of byproducts, such as higher alcohols, esters, acids, aldehydes, ketones and terpenes. These metabolites contribute to the fragrance of tangerine wines [5,6,11]. Likewise, esters and higher alcohols were found to be the primary fragrance components in tangerine wines (Figure S1). These are formed during alcoholic fermentation and contribute significantly to the taste of wine [29].

Higher alcohols are produced through the Erlich pathway in the presence of amino acids by yeasts during alcoholic fermentation [19]. Higher alcohols are the most abundant group of volatile chemicals in several kinds of tangerine wines, and they could contribute favorably to the wine’s fresh fruity taste, vegetal notes and olfactory complexity [48]. While higher alcohols may react with organic acids to generate esters with a pleasant taste, excessive concentrations (500 mg/L) may result in an unpleasant flavor in alcoholic beverages [6]. In this study, the concentrations of higher alcohols in all fermentations were less than 500 mg/L, which means that a higher alcohol content might not result in an undesirable flavor in tangerine wine. However, excessive concentrations of higher alcohols (580.72 mg/L) were reported in *Torulaspora delbrueckii*/*S. cerevisiae* sequential fermentation [6]. This disparity might be explained by the strains used, indicating that there are significant differences in the synthesis of higher alcohols across different yeasts and fermentation techniques. Among the higher alcohol groups, phenylethanol with a faint rose scent and 1-decanol with a fruity, sweet, floral, unique fatty aroma were the most active odorants (OAV ≥ 1) [11,20] (Table S4). The phenylethanol concentrations in most co-fermentations were greater than that produced with pure Sc fermentation. In particular, Hg-10-Sc sequential fermentation produced the greatest quantity of phenylethanol. The results are in agreement with previous reports [6]; however, the phenylethanol content of the tangerine wines fermented in this study (197.23 mg/L) was significantly higher than that described in a previous report (33.21 mg/L) [6], which may be related to the fermentation process.

Esters may lend fruity and flowery aromas to fruit wines [49]. Isoamyl acetate, ethyl hexanoate, ethyl octanoate, phenethyl acetate, ethyl decanoate and ethyl laurate were the primary esters found in tangerine wines. The contents of ester compounds in the majority of co-fermentations were higher than in pure Sc fermentation (Figure S1). In terms of the volatile odor-active compounds (OAV ≥ 1) present in tangerine wines (Table S4), ester compounds, particularly ethyl octanoate, ethyl hexanoate and isoamyl acetate, contribute more to tangerine wine fragrance than higher alcohols. Ethyl octanoate has a fruity banana scent; ethyl hexanoate has a fruity, wine-like perfume; and isoamyl acetate has a banana-pear aroma [50].

As shown in Table S3, only the sequential fermentations with Hg-10-Sc and Ce-10-Sc yielded octanoic acid, the only volatile acid. Octatonic acid was the sole odor-active fatty acid detected in Hg-10-Sc sequential fermentation (OAV = 1.33), as shown in Table S4. Octanoic acid is often associated with a cheesy odor and rotten smell. However, at low concentrations, octanoic acid possesses a pleasant fruity aroma. The high content of octatonic acid in Hg-10-Sc sequential fermentation (11.71 mg/L) was consistent with that described in a previous report, which reported that Sc-Td co-fermentation greatly increased the octatonic acid (20.04 mg/L) content, but an excessive content of fatty acids (≥20 mg/L) might produce a rancid flavor [6].

While higher alcohols, esters and fatty acids were discovered to be the most abundant volatile components in tangerine wines, volatile aldehydes, ketones, terpenes and phenols were also detected. These greatly promote the flavor complexity of tangerine wines as well. Numerous terpenes and aldehyde compounds, including D-limonene, linalool, terpineol, citronellol and decanal, have been detected in tangerine wines, which is consistent with previous reports [3,5,6]. Terpenols are often connected with floral and tangerine scents; therefore, it is reasonable to assume that wines with greater terpenol concentrations would have stronger flowery and citrus scents [51]. The contents of terpenoids in Ht-10-Sc, Sc:Ce-1:1 and Sc:Hg-1:100 fermentations were significantly higher than those in other fermentations, indicating that these three fermentations retained the tangerine wine flavor better.

### 3.4. Principal Component Analysis of Volatile Aroma Compounds (OAV ≥ 1) and Electronic Nose Data in Tangerine Wine

**Figure 6:**
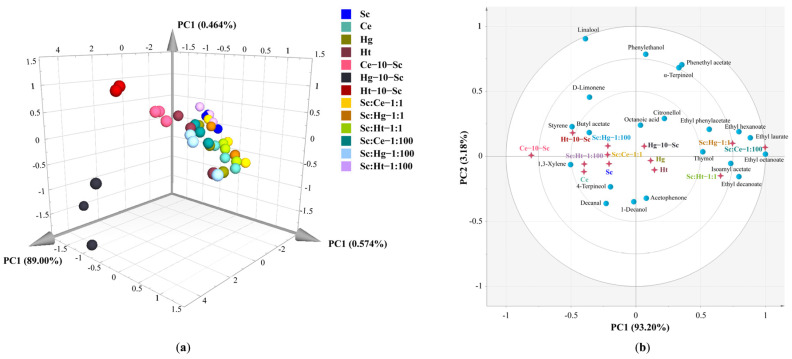
The principal component analysis: (**a**) E-nose datasets; (**b**) volatile aroma compounds (OAV ≥ 1). Abbreviations: Sc, *S. cerevisiae*; Ce, *C. ethanolica*; Hg, *H. guilliermondii*; Ht, *H. thailandica*.

To further investigate the correlation and segregation of different fermentation methods on volatile aroma compounds, the results of volatile components (OAV ≥ 1) and the electronic nose program were analyzed by a principal component analysis. The results show that Hg-10-Sc sequential fermentation led to substantial differences compared with all other tangerine wine processes (Figure 6a). The PCA analysis of active odor compounds (OAV ≥ 1) in tangerine wines (Figure 6b) showed that the Hg-10-Sc sequential fermentation significantly clustered with octanoic acid, which was characterized by “rancid, harsh, cheese, fatty acid”. Octanoic acid, in particular, possesses a pleasant fruity aroma at low concentrations, and it was the only odor-active fatty acid present in wines produced through Hg-10-Sc sequential fermentation. Simultaneously, Ce-10-Sc sequential fermentation significantly clustered with two active odor compounds: styrene with a sweet fruit aroma and butyl acetate with a fruity aroma. These results highlight that different fermentation strategies are associated with different potential aroma biomarkers and different flavor characteristics in tangerine wines, which is consistent with previous reports [6,8,36].

### 3.5. Other Metabolites

#### 3.5.1. Ethanol

In pure fermentation, the ethanol percentage of tangerine wines prepared with only a non-*Saccharomyces* yeast was lower (7.79 percent–9.39 percent) than in wines made entirely with Sc (11.13 percent). Pure Ce fermentation (9.39 percent) produced wine containing a significantly higher concentration of ethanol than that produced through pure fermentation with Hg (8.06 percent) and Ht (7.79 percent). Additionally, pure Ce fermentation produced wine with a lower concentration of residual sugar (1.92 g/L), while pure fermentation with Hg (2.75 g/L) and Ht (2.48 g/L) produced wine with more residual sugar (Table 5). These results are in agreement with those of previous reports showing lower overall ethanol yields in wines produced with non-*Saccharomyces* yeasts than in those produced with Sc because of the greater yeast biomass or byproduct formation from the consumption of sugars [8,52]. In wines produced through co-fermentation, there was no significant difference in ethanol concentration between those produced through pure Sc fermentation compared with those produced through sequential fermentations, but the ethanol content declined dramatically with other fermentations. These results are in agreement with the results of previous reports [8,36], while other reports have shown that wines produced through sequential fermentations have a lower content of ethanol than those produced through pure Sc fermentation [6]. This might be due to the use of different fruit juices, fermentation strategies and yeasts.

Sequential fermentations produced higher ethanol concentrations by digesting more sugar. However, wines with a high alcohol content may be viewed negatively due to health concerns, reduced wine flavor and tax rates based on alcohol content [52,53,54,55]. As a result, tangerine wines with a low ethanol percentage are more marketable and consumer-friendly. If the next product is tangerine vinegar, we can use tangerine wines with a greater ethanol level for acetic acid fermentation, resulting in tangerine vinegar with a high acetic acid concentration.

#### 3.5.2. Organic Acids

Organic acids are critical in numerous parts of the wine-making process, as acidity impacts a wine’s flavor and color intensity [56]. Esters are thought to be formed predominantly as a result of alcohols being esterified with organic acids during the fermentation and storage processes, imparting fruity and floral aromas to wines [29]. As a result, organic acids may play a role in the tangerine wine’s complex aroma. These distinctions are probably due to yeasts employing separate processes for generating and decomposing organic acids [18]. In this study, the concentration of lactic acid in non-*Saccharomyces* yeast fermentations was significantly higher than that in pure Sc fermentations (Table 5). Lactic acid is formed when alcoholic fermentation is followed by malolactic fermentation, in which the acidic malic acid is converted to the softer lactic acid [57]. Lactic acid concentrations above a certain level also result in wines with a softer flavor and lower acidity [11]. Tartaric and malic acids play significant roles in determining the acidity of wine [36]. In comparison to pure fermentation, the lower malic acid level produced from co-fermentation may dramatically lessen the acidity of tangerine wines. Furthermore, fumaric acid was only presented in tangerine juice with astringent and acidic taste, indicating that alcoholic fermentation positively influences the mouthfeel perception of wine. Interestingly, acetic acid was detected exclusively in tangerine juice and tangerine wines produced by pure fermentation with Sc. In comparison with co-fermentation, pure Sc fermentation may have consumed more citric acid, obtaining a higher acetic acid output [58]. No acetic acid was detected in the co-fermentations, possibly due to lower citric acid consumption, which would have resulted in less acetic acid production. Additionally, acetic acid is thought to improve the wine flavor profile as the precursor of fruity acetate esters via acetyl-CoA, but concentrations above 0.7 g/L result in an unpleasant odor and taste [58]. In this study, the concentrations of acetic acid in tangerine juice and tangerine wines produced through pure Sc fermentation were 0.49 g/L and 5.08 g/L, respectively (Table 5), indicating that pure Sc fermentation may result in tangerine wines with an unattractive taste. In general, fermentations involving non-*Saccharomyces* yeasts produced significantly more lactic acid and no acetic acid, indicating that non-*Saccharomyces* yeasts have a beneficial effect on tangerine wine flavor.

## 4. Discussion

This comprehensive study provides new insights into the selection of suitable co-fermentation methods with non-*Saccharomyces* yeasts and Sc for the production of tangerine wines. In terms of pure fermentations, Ce showed the best polyphenol production ability. Pure fermentation with Hg produced a higher level of volatile aroma components than pure fermentation with other yeasts. In terms of co-fermentations, the polyphenols content produced through sequential fermentation with Hg and Ce was significantly higher than that produced with other fermentations. Fermentation with Sc:Hg-1:1 (588.35 mg/L) produced the maximum amount of volatile aroma compounds as well as a lower ethanol concentration (8.30%), which is more in line with consumer demands for rich flavor and a low alcohol concentration. The Hg-10-Sc sequential fermentations generated higher ethanol concentrations (10.73%), more total polyphenols (140.24 mg/L), a high amount of volatile aroma compounds (518.66 mg/L) and more complex aromas. Simultaneously, Hg-10-Sc sequential fermentation imparted more pronounced honey, floral aroma and a spicy flavor because of having the highest phenylethanol concentration. Octanoic acid, in particular, was the only odor-active fatty acid present in wine produced through Hg-10-Sc sequential fermentation and contributed to a distinct aroma in tangerine wine. In general, the wine produced through Hg-10-Sc sequential fermentation met consumer demands for higher biological activity, a higher aroma complexity and a distinctive tangerine wine flavor. The mechanism by which sequential fermentation can improve the content of polyphenols and the sensory quality of tangerine wines is not clear. Additionally, the quality of 1:100 mixed fermentation tangerine wines was not ideal. Thus, further research into the molecular processes responsible for non-*Saccharomyces* and *S. cerevisiae* metabolic profiles in co-fermentation systems is warranted to offer more information on the mechanisms underlying non-*Saccharomyces* and *S. cerevisiae* as well as the connections between fermentation products and strains.

## Figures and Tables

**Table 1:** The fermentation groups.

Pure Fermentation	Sequential Fermentation	Mixed Fermentation (1:1)	Mixed Fermentation (1:100)
Sc	Ce-10-Sc	Sc:Ce-1:1	Sc:Ce-1:100
Ce	Hg-10-Sc	Sc:Hg-1:1	Sc:Hg-1:100
Hg	Ht-10-Sc	Sc:Ht-1:1	Sc:Ht-1:100
Ht			

Abbreviations: Sc, *S. cerevisiae*; Ce, *C. ethanolica*; Hg, *H. guilliermondii*; Ht, *H. thailandica*.

**Table 2:** Mobile phase gradient elution procedure.

Time (min)	0	2	10	12	15	20	25	30
0.3% acetic acid solution	80	80	75	70	60	50	40	80
Acetonitrile	20	20	25	30	40	50	60	20

**Table 3:** Mobile phase gradient elution procedure.

Time (min)	0	10	20	25	35
2% acetic acid solution	85	80	80	70	85
Methanol	15	20	20	30	15

**Table 4:** Chemical sensors used in the electronic nose program corresponding to different types of volatile substances.

Sensor Number	Sensor Name	Sensor Sensitive Compounds
1	T70/2	Aromatic compounds
2	PA/2	Ethanol, aromatic/organic amine
3	P30/1	Hydrocarbons, ammonia, ethanol
4	P40/1	Chlorine
5	LY2/AA	Ammonia/organic amine
6	LY2/gCT	Hydrogen sulfide

**Table 5:** The content of ethanol and organic acids in tangerine wines.

	Ethanol (%)	Organic Acids (mg/L)									
		Oxalic Acid	Malic Acid	Vitamin C	Lactic Acid	Acetic Acid	Mleic Acid	Citric Acid	Succinic Acid	Fumaric Acid	Subtotal
Juice	-	877.29 ± 3.14a	103.07 ± 6.48f	119.30 ± 1.52de	517.54 ± 3.51h	493.93 ± 45.52b	-	8165.07 ± 11.51a	249.76 ± 12.46g	3.17 ± 0.09	10,529.12 ± 62.41f
Sc	11.13 ± 0.02a	536.36 ± 57.36bc	230.77 ± 3.48b	135.29 ± 2.03bc	5949.32 ± 22.39g	5084.68 ± 57.57a	-	5789.19 ± 170.51cd	1976.67 ± 15.41bc	-	19,702.29 ± 285.60a
Ce	9.39 ± 0.32b	396.83 ± 44.51g	228.65 ± 28.48b	149.29 ± 9.05a	8338.23 ± 81.41bc	-	-	5795.31 ± 119.19cd	1783.86 ± 149.45de	-	16,692.17 ± 268.74cde
Hg	8.06 ± 0.53cde	516.91 ± 26.95cd	274.74 ± 3.48a	112.00 ± 15.40e	7908.25 ± 19.94de	-	-	5858.49 ± 4.67cd	1566.96 ± 15.58f	-	16,237.34 ± 58.42e
Ht	7.79 ± 0.99de	585.88 ± 30.59b	263.85 ± 4.48a	137.75 ± 3.72bc	7947.0 ± 201.50de	-	-	5820.35 ± 116.41cd	1633.81 ± 144.50ef	-	16,388.66 ± 321.33de
Ce-10-Sc	10.74 ± 0.03a	455.92 ± 26.31defg	183.08 ± 1.48d	135.44 ± 2.28bc	8016.5 ± 211.11cde	-	-	6372.43 ± 118.01b	1935.11 ± 203.89bcd	-	17,098.58 ± 272.01bc
Hg-10-Sc	10.73 ± 0.08a	414.59 ± 5.25efg	176.40 ± 11.48d	126.87 ± 1.55cd	7739.8 ± 42.11ef	-	-	6549.95 ± 45.93b	2317.06 ± 17.34a	-	17,324.69 ± 91.46b
Ht-10-Sc	10.81 ± 0.03a	463.41 ± 5.05def	132.56 ± 0.48e	140.64 ± 0.62ab	7457.5 ± 86.81f	-	-	6471.23 ± 19.22b	2106.61 ± 35.22b	-	16,772.02 ± 137.77cd
Sc:Ce-1:1	8.59 ± 0.14bcd	464.27 ± 5.35def	203.76 ± 4.48c	133.99 ± 3.95bc	8659.1 ± 204.95ab	-	-	5679.36 ± 47.04d	1774.93 ± 41.08de	-	16,915.48 ± 223.27bc
Sc:Hg-1:1	8.30 ± 0.36cd	504.22 ± 25.28cd	184.10 ± 0.48d	127.63 ± 1.69cd	8566.4 ± 64.93ab	-	-	5829.65 ± 31.74cd	1704.18 ± 29.04ef	-	16,916.20 ± 85.11bc
Sc:Ht-1:1	8.35 ± 0.43cd	485.14 ± 58.24cd	177.64 ± 2.48d	141.88 ± 2.11ab	8723.6 ± 438.83a	-	-	5927.20 ± 149.75c	1914.85 ± 89.35cd	-	17,370.35 ± 468.01b
Sc:Ce-1:100	7.39 ± 0.57e	410.27 ± 0.62fg	140.13 ± 5.48e	135.40 ± 3.57bc	8103.1 ± 28.75cd	-	-	5713.95 ± 24.61d	1926.10 ± 15.77cd	-	16,429.00 ± 54.33de
Sc:Hg-1:100	8.62 ± 0.26bcd	476.75 ± 9.26cde	132.96 ± 3.48e	140.37 ± 4.55ab	8061.8 ± 71.21cde	-	-	5847.13 ± 9.44cd	2007.81 ± 14.64bc	-	16,666.88 ± 80.42cde
Sc:Ht-1:100	8.85 ± 0.28bc	503.82 ± 32.29cd	132.21 ± 0.48e	135.65 ± 4.10bc	8158.0 ± 164.32cd	-	-	5918.53 ± 108.60c	2062.12 ± 35.67bc	-	16,910.34 ± 263.76bc

## Data Availability

All data generated or analyzed in this study are available within the manuscript and are available from the corresponding authors upon request.

## Supplementary Materials

The following supporting information can be downloaded at: https://www.mdpi.com/article/10.3390/jof8020128/s1, Figure S1: The concentration of volatile aroma compounds identified in tangerine wines. Table S1. Chemical composition of tangerine juice and tangerine wines (means ± SD). Table S2: The standard curve, validation range and coefficient of determination (r2) for the non-volatile aroma compounds in tangerine wines. Table S3: Volatile aroma compounds (mg/L) from tangerine wines. Table S4: Volatile odor-active compounds (OAV ≥ 1) in tangerine wines.
