# Supplementary material for: Use of Non-Saccharomyces Yeast Co-Fermentation with Saccharomyces cerevisiae to Improve the Polyphenol and Volatile Aroma Compound Contents in Nanfeng Tangerine Wines
Source: J Fungi (Basel). 2022 Jan 27;8(2):128. doi: 10.3390/jof8020128 (PMC8875693; doi:10.3390/jof8020128)
Supplement: Supplementary file 1 [file jof-08-00128-s001.zip › Table S1.pdf]

Table S1. Chemical composition of tangerine juice and tangerine wines.

	**Juice**	**Sc**	**Ce**	**Hg**	**Ht**	**Ce-10-Sc**	**Hg-10-Sc**	**Ht-10-Sc**	**Sc:Ce-1:1**	**Sc:Hg-1:1**	**Sc:Ht-1:1**	**Sc:Ce-1:100**	**Sc:Hg-1:100**	**Sc:Ht-1:100**
**Reducing sugar (g/L)**	-	1.56±0.13f	1.92±0.04ef	2.75±0.05b	2.48±0.03bc	0.87±0.06g	2.33±0.08cd	1.00±0.13g	2.05±0.21de	2.43±0.46bc	2.24±0.19cde	2.05±0.15de	2.22±0.12cde	3.27±0.23a
**pH**	4.13±0.02cd	4.14±0.01c	4.20±0.01a	4.17±0.01b	4.18±0.02ab	4.03±0.00i	3.96±0.00j	4.00±0.01k	4.11±0.01de	4.12±0.00cde	4.05±0.00hi	4.06±0.01gh	4.10±0.03ef	4.08±0.00fg
**Total acidity (g/L)**	8.96±0.16g	9.97±0.24ef	10.51±0.09bcd	10.40±0.16bcd	10.77±0.18b	10.35±0.18cde	11.25±0.09a	10.72±0.28bc	10.61±0.24bcd	9.81±0.24f	10.24±0.32de	9.97±0.09ef	10.29±0.09de	10.72±0.16bc
**flavonoids (mg/L)**														
**Eriocitrin**	3.11±0.01h	3.46±0.01a	3.42±0.00bc	3.39±0.02cde	3.36±0.03fg	3.45±0.02ab	3.47±0.02a	3.47±0.01a	3.41±0.02cd	3.33±0.00g	3.38±0.01def	3.38±0.02ef	3.39±0.01cde	3.39±0.00cde
**Narirutin**	24.08±0.01e	24.93±0.54d	26.08±0.23ab	26.34±0.47a	25.52±0.59bcd	26.13±0.08ab	26.35±0.29a	25.49±0.04bcd	25.51±0.21bcd	23.95±0.52e	25.26±0.22cd	25.57±0.12bcd	25.78±0.23abc	25.61±0.36bcd
**Hesperidin**	34.98±0.11def	36.45±0.84abc	37.16±0.29a	36.56±0.52ab	35.33±0.77cdef	35.70±0.82bcde	35.77±0.44bcd	34.71±0.06def	35.37±0.06bcdef	32.97±0.37g	34.36±0.10f	34.50±1.21ef	34.90±0.76def	34.42±0.30f
**Neohesperidin**	5.21±0.06a	4.47±0.03ef	4.43±0.04efg	4.43±0.03efg	4.48±0.01de	4.63±0.07c	4.81±0.01b	4.80±0.01b	4.58±0.13cd	4.36±0.01g	4.39±0.05fg	4.34±0.01g	4.37±0.03g	4.39±0.02efg
**Didymin**	4.64±0.01d	4.82±0.05b	4.82±0.01b	4.78±0.04bc	4.74±0.03c	4.91±0.02a	4.84±0.03b	4.83±0.01b	4.73±0.02c	4.64±0.01d	4.72±0.02c	4.72±0.05c	4.73±0.03c	4.72±0.01c
**Hesperetin**	-	3.86±0.03a	3.51±0.06b	3.21±0.21fg	3.25±0.06efg	3.71±0.10a	3.35±0.01cdef	3.50±0.01bc	3.43±0.04bcd	3.17±0.06g	3.33±0.05def	3.46±0.00bcd	3.39±0.06bcde	3.43±0.01bcd
**Nobiletin**	-	0.90±0.01bc	0.93±0.01b	0.87±0.04de	0.86±0.01de	1.02±0.01a	0.91±0.02b	0.92±0.00b	0.79±0.02f	0.79±0.01f	0.85±0.01e	0.88±0.01cd	0.86±0.01de	0.92±0.01b
**Subtotal**	72.02±0.14d	78.90±1.25ab	80.00±0.70a	79.60±0.08a	77.32±1.42bc	79.54±0.93a	79.51±0.49a	77.72±0.01bc	77.55±0.36bc	73.43±0.87d	76.16±0.26c	76.76±1.33c	77.54±0.76bc	76.39±0.56c
**phenolic acids (mg/L)**														
**Protocatechuic acid**	3.15±0.00cd	3.40±0.04a	3.02±0.03e	2.99±0.02ef	3.03±0.06e	3.17±0.09c	2.89±0.02f	3.32±0.01ab	3.34±0.02ab	3.39±0.05a	3.35±0.02ab	3.04±0.01de	3.17±0.13c	3.26±0.10bc
**p-Hydroxybenzoic acid**	1.63±0.00g	2.47±0.00a	2.23±0.01de	2.21±0.03e	2.19±0.04e	2.30±0.06c	2.33±0.05bc	2.23±0.01de	2.35±0.02bc	2.37±0.01b	2.32±0.04bc	2.10±0.02f	2.29±0.01cd	2.29±0.01cd
**Chlorogenic acid**	17.38±0.02f	17.69±0.03ef	18.49±0.31c	18.08±0.11cde	18.21±0.27cd	19.26±0.02b	19.91±0.18a	19.88±0.10a	18.48±0.25c	17.75±0.43def	18.41±0.54c	18.38±0.02c	18.21±0.14cd	18.18±0.04cd
**Caffeic acid**	17.65±0.02a	15.66±0.04e	16.85±0.24bcd	16.60±0.01cd	16.65±0.39cd	16.99±0.23bc	16.92±0.29bcd	16.75±0.08bcd	16.58±0.00cd	15.90±0.09e	16.69±0.09bcd	17.12±0.50b	16.66±0.16cd	16.55±0.08d
**p-Coumaric acid**	9.75±0.02abc	9.69±0.04bcd	9.96±0.16a	9.70±0.02bcd	9.92±0.23ab	9.92±0.22ab	9.42±0.03e	9.47±0.02de	9.88±0.18ab	9.48±0.00de	9.62±0.05cde	9.48±0.01de	9.60±0.11cde	9.45±0.14e
**Ferulic acid**	3.91±0.01a	3.04±0.08ef	2.97±0.04fg	3.51±0.27bc	3.35±0.05cd	3.19±0.02de	3.58±0.07b	3.23±0.02d	2.90±0.12fgh	2.97±0.02fg	2.98±0.10fg	2.78±0.02h	2.83±0.01gh	2.91±0.01fgh
**Sinapic Acid**	4.96±0.01e	5.60±0.12bc	5.26±0.02d	5.54±0.20c	5.31±0.02d	5.83±0.01a	5.69±0.05ab	5.68±0.02bc	5.24±0.02d	5.26±0.07d	5.21±0.02d	5.22±0.01d	5.22±0.02d	5.21±0.01d
**Subtotal**	58.44±0.03bcd	57.56±0.20ef	58.78±0.58b	58.62±0.40bc	58.67±0.62bc	60.67±0.49a	60.73±0.29a	60.56±0.07a	58.77±0.03b	57.12±0.48f	58.57±0.41bcd	58.12±0.49bcde	57.97±0.10cde	57.84±0.13def
**Total polyphenols (mg/L)**	130.46±0.12f	136.47±1.44cd	138.78±1.25ab	138.22±0.39bc	135.99±1.89de	140.21±0.90a	140.24±0.56a	138.27±0.07bc	136.32±0.39d	130.56±0.63f	134.73±0.17de	134.89±1.15de	135.51±0.75de	134.23±0.47e

Abbreviations: Sc, *S. cerevisiae*; Ce, *Candida ethanolica*; Hg, *Hanseniaspora guilliermondii*; Ht, *Hanseniaspora thailandica*; “-” presented no detection. Data shown are the mean ± SD of triplicate, values with different Roman letters in the same row indicated significantly different at $P < 0.05$ (Duncan's test).
