# Supplementary material for: Use of Non-Saccharomyces Yeast Co-Fermentation with Saccharomyces cerevisiae to Improve the Polyphenol and Volatile Aroma Compound Contents in Nanfeng Tangerine Wines
Source: J Fungi (Basel). 2022 Jan 27;8(2):128. doi: 10.3390/jof8020128 (PMC8875693; doi:10.3390/jof8020128)
Supplement: Supplementary file 1 [file jof-08-00128-s001.zip › Table S2.pdf]

Table S2. The standard curve, validation range and coefficient of determination (r^2) for the non-volatile aroma compounds in tangerine wines.

No	Compounds	Standard curve	r^2	Validation range (mg/L)
	**Organic acids**			
1	Oxalic acid	y = 5.5678x + 13.608	0.9982	5-6120
2	Malic acid	y = 0.7528x - 1.7245	0.9999	20-1000
3	Vitamin C	y = 8.5032x - 108.46	0.9999	25-1000
4	Lactic acid	y = 0.3335x - 2.2668	0.9999	25-17000
5	Acetic acid	y = 0.3943x - 14.812	0.9998	50-7480
6	Maleic acid	y = 82.9x + 5.5872	0.9999	5-100
7	Citric Acid	y = 0.8067x + 16.424	0.9999	50-10000
8	Succinic acid	y = 0.4433x - 1.0015	0.9999	25-3200
9	Fumaric acid	y = 112x - 131.65	0.9980	1-50
	**Phenolic acids**			
10	Protocatechuic acid	y = 32.7x - 42.145	0.9999	1.5-200
11	p-Hydroxybenzoic acid	y = 54.729x - 75.537	0.9989	0.5-150
12	Chlorogenic acid	y = 19.026x - 122.05	0.9985	2.5-250
13	Caffeic acid	y = 57.802x - 134.69	0.9997	2-200
14	p-Coumaric acid	y = 68.565x - 147.67	0.9997	2-200
15	Ferulic acid	y = 47.436x - 89.625	0.9995	0.5-200
16	Sinapic Acid	y = 36.101x - 140.13	0.9985	1-250
	**flavonoids**			
17	Eriocitrin	y = 27.672x - 40.059	0.9985	1-150
18	Narirutin	y = 13.859x - 5.4434	0.9998	1-100
19	Hesperidin	y = 15.409x + 68.192	0.9983	1-500
20	Neohesperidin	y = 18.133x - 26.053	0.9983	1-107
21	Didymin	y = 18.994x - 52.254	0.9903	1-117.5
22	Hesperetin	y = 18.319x - 15.997	0.9994	1-100
23	Nobiletin	y = 38.344x + 2.2137	0.9991	0.5-170
