# Supplementary material for: Use of Non-Saccharomyces Yeast Co-Fermentation with Saccharomyces cerevisiae to Improve the Polyphenol and Volatile Aroma Compound Contents in Nanfeng Tangerine Wines
Source: J Fungi (Basel). 2022 Jan 27;8(2):128. doi: 10.3390/jof8020128 (PMC8875693; doi:10.3390/jof8020128)
Supplement: Supplementary file 1 [file jof-08-00128-s001.zip › Table S3.pdf]

Table S3. Volatile aroma compounds (mg/L) from tangerine wines.

**Compounds** [a]	**Sc**	**Ce**	**Hg**	**Ht**	**Ce-10-Sc**	**Hg-10-Sc**	**Ht-10-Sc**	**Sc:Ce-1:1**	**Sc:Hg-1:1**	**Sc:Ht-1:1**	**Sc:Ce-1:100**	**Sc:Hg-1:100**	**Sc:Ht-1:100**
**Higher alcohols**													
1-Octanol	- [b]	-	1.28±0.05a [c]	0.97±0.19bc	0.95±0.05c	-	-	-	-	-	-	-	1.03±0.11b
2,3-Butanediol	-	-	-	-	-	-	-	-	-	-	-	3.28±0.10	-
Phenylethanol	197.23±197.23h	212.01±0.50f	222.96±0.72e	169.20±30.50i	180.70±0.72i	301.02±2.07a	284.29±2.74bc	248.61±1.48d	285.58±1.74b	196.57±1.40h	215.33±1.53f	281.20±1.65c	206.11±1.72g
1-Decanol	1.53±1.53a	-	1.17±0.02b	0.89±0.16c	-	-	-	-	-	-	-	-	-
**Esters**													
Butyl acetate	-	1.28±0.07e	-	-	1.81±0.00c	-	2.23±0.11b	-	1.62±0.04d	-	-	-	3.07±0.03a
Isoamyl acetate	7.92±7.92i	7.14±0.03j	34.17±0.07f	55.32±12.32b	7.55±0.07ij	36.07±0.21e	19.49±0.28g	10.01±0.02h	51.17±0.22c	72.48±0.45a	40.83±0.33d	2.38±0.00l	6.26±0.02k
Ethyl hexanoate	1.36±1.36h	0.98±0.07j	12.05±0.06c	11.74±0.17d	1.12±0.06i	11.25±0.03e	2.45±0.05f	1.80±0.06g	12.17±0.03b	11.85±0.03d	17.52±0.07a	11.35±0.20e	1.17±0.08i
Octyl formate	1.66±1.66b	-	-	-	5.75±0.00a	-	-	-	-	-	1.25±0.02d	1.39±0.06c	-
4-Tolyl isocyanate	-	-	-	-	1.84±0.00c	6.17±0.06a	-	-	-	-	-		1.98±0.08b
Ethyl octanoate	7.59±7.59h	5.12±0.03i	11.76±0.03e	12.11±0.25d	-	11.22±0.14f	4.33±0.06j	7.64±0.01gh	19.97±0.13b	18.59±0.15c	23.21±0.12a	7.79±0.12g	5.25±0.08i
Ethyl phenylacetate	-	-	-	-	-	-	-	-	-	-	1.17±0.03	-	-
Phenethyl acetate	43.75±43.75j	48.25±0.08i	51.51±0.13h	42.34±5.16k	47.80±0.13i	72.02±0.54c	78.92±0.83b	58.58±0.30f	100.55±0.62a	62.64±0.47e	57.13±0.39g	65.04±0.16d	51.29±0.47h
Ethyl decanoate	22.37±22.37c	15.49±0.00f	15.40±0.02f	15.28±0.05f	9.05±0.02g	18.89±0.18e	8.78±0.02h	19.52±0.04d	26.34±0.22a	24.57±0.18b	24.37±0.13b	7.61±0.07i	8.97±0.13gh
Ethyl laurate	4.42±4.42h	5.11±0.03f	5.56±0.02e	4.61±0.54g	2.72±0.02i	6.26±0.11d	5.03±0.05f	4.68±0.05g	11.87±0.11a	8.92±0.09c	11.55±0.03b	1.56±0.04j	2.77±0.13i
**Terpenes**													
Linalool	5.17±5.17f	4.95±0.03g	5.32±0.02f	4.35±0.55h	7.63±0.02b	7.67±0.02b	9.71±0.03a	6.00±0.02e	6.87±0.01d	2.25±0.03i	5.91±0.02e	7.12±0.19c	5.18±0.12f
4-Terpineol	12.30±12.30e	12.43±0.08e	13.74±0.01cd	11.95±1.03f	8.44±0.01h	10.05±0.03g	12.04±0.21f	15.30±0.03b	-	13.65±0.15d	13.96±0.08c	17.44±0.13a	12.05±0.12f
α-Terpineol	17.84±17.84h	19.80±0.06f	21.52±0.04e	17.07±2.53i	16.40±0.04j	21.31±0.23e	23.28±0.32c	22.28±0.07d	25.44±0.22a	18.76±0.14g	21.45±0.12e	24.78±0.24b	14.74±0.16k
Citronellol	1.23±1.23c	1.05±0.04d	-	-	-	-	1.76±0.06b	-	-	-	2.82±0.04a	-	-
Styrene	12.46±12.46d	8.30±0.04f	-	-	-	-	15.84±0.10c	24.05±0.10a	-	-	-	19.76±0.02b	10.52±0.04e
D-Limonene	6.25±6.25f	4.90±0.06i	5.77±0.03g	6.23±0.28f	6.40±0.03f	2.69±0.02j	13.41±0.08b	11.06±0.02c	7.37±0.02e	5.63±0.02g	5.24±0.02h	14.78±0.12a	9.11±0.01d
Acetophenone	-	-	-	0.88±0.52	-	-	-	-	-	-	-	-	-

Table S3. Cont.

Compounds	Sc	Ce	Hg	Ht	Ce-10-Sc	Hg-10-Sc	Ht-10-Sc	Sc:Ce-1:1	Sc:Hg-1:1	Sc:Ht-1:1	Sc:Ce-1:100	Sc:Hg-1:100	Sc:Ht-1:100
**Aldehydes and ketones**													
Decanal	-	1.06±0.03	-	-	-	-	-	-	-	-	-	-	-
2,5-Dimethylbenzaldehyde	1.48±1.48e	2.73±0.04c	5.43±0.02a	-	-	-	-	2.24±0.05d	4.63±0.06b	1.47±0.04e	1.27±0.03f	-	-
2-Hydroxy-5-methylacetophenone	12.28±12.28h	17.52±0.02d	17.69±0.09c	15.28±1.36g	-	-	-	16.28±0.04f	22.33±0.18a	16.64±0.15e	20.28±0.10b	-	-
**Phenols**													
Thymol	5.47±5.47c	5.88±0.01b	5.78±0.01b	4.22±0.89h	3.11±0.01i	4.76±0.06d	4.48±0.03f	4.42±0.01fg	6.36±0.09a	4.53±0.09ef	5.38±0.01c	4.64±0.01de	4.33±0.12gh
2,4-Ditert-butylphenol	-	-	1.21±0.01a	0.94±0.15b	-	-	-	-	-	-	-	-	-
**Acids**													
Octanoic acid	-	-	-	-	5.23±0.00b	11.71±0.15a	-	-	-	-	-	-	-
**Other compounds**													
1,3-Xylene	3.84±3.84c	2.82±0.06e	2.37±0.03g	-	2.53±0.03f	-	-	6.22±0.02b	-	-	-	7.25±0.11a	3.54±0.07d
1-Methyl-3-isopropylbenzene	-	-	-	1.28±0.72a	-	-	-	-	-	1.17±0.03b	1.10±0.05c	-	-
1-Isopropenyl-3-methylbenzene	1.11±1.11d	1.04±0.02d	1.79±0.04a	1.46±0.20c	-	-	-	-	1.57±0.07b	1.61±0.06b	1.43±0.02c	-	-
4-Methyl-1,5-dioxaspiro[5.5]undecane	4.17±4.17g	4.71±0.01e	5.87±0.00c	5.92±0.06c	-	-	-	3.80±0.06h	4.51±0.11f	4.94±0.11d	6.40±0.02b	11.50±0.00a	-
4-Hydroxy-3-Methoxybenzonitrile	-	-	-	-	19.94±0.00c	28.23±0.25b	32.60±0.27a	-	-	-	-	-	18.37±0.20d
**Total**	371.41±371.41i	382.55±0.90h	442.34±1.11g	382.03±33.83h	328.95±1.11j	549.31±4.03b	518.66±4.98c	462.50±1.78f	588.35±3.61a	466.29±3.33f	477.61±2.72e	488.88±3.04d	365.73±3.29i

Abbreviations: Sc, *S. cerevisiae*; Ce, *Candida ethanolica*; Hg, *Hanseniaspora guilliermondii*; Ht, *Hanseniaspora thailandica*; "-" presented no detection. Data shown are the mean ± SD of triplicate, values with different Roman letters in the same row indicated significantly different at P < 0.05 (Duncan's test).
