# Supplementary material for: Use of Non-Saccharomyces Yeast Co-Fermentation with Saccharomyces cerevisiae to Improve the Polyphenol and Volatile Aroma Compound Contents in Nanfeng Tangerine Wines
Source: J Fungi (Basel). 2022 Jan 27;8(2):128. doi: 10.3390/jof8020128 (PMC8875693; doi:10.3390/jof8020128)
Supplement: Supplementary file 1 [file jof-08-00128-s001.zip › Table S4.pdf]

Table S4. Volatile odor-active compounds (OAV ≥ 1) in tangerine wines.

**Compounds**	**Threshold μg/L** [a]	**Description** [b]	**Sc**	**Ce**	**Hg**	**Ht**	**Ce-10-Sc**	**Hg-10-Sc**	**Ht-10-Sc**	**Sc:Ce-1:1**	**Sc:Hg-1:1**	**Sc:Ht-1:1**	**Sc:Ce-1:100**	**Sc:Hg-1:100**	**Sc:Ht-1:100**
**Phenylethanol**	14.00	Rose	14.09 [c]	15.14	15.93	12.09	12.91	21.50	20.31	17.76	20.40	14.04	15.38	20.09	14.72
**1-Decanol**	0.50	Fruit, sweet, flower, special fatty	3.07	< 1	2.34	1.78	< 1	< 1	< 1	< 1	< 1	< 1	< 1	< 1	< 1
**Butyl acetate**	1.83	Fruit	< 1	< 1	< 1	< 1	< 1	< 1	1.22	< 1	< 1	< 1	< 1	< 1	1.68
**Isoamyl acetate**	0.03	Banana, pear	263.95	237.85	1138.83	1844.10	251.70	1202.46	649.72	333.80	1705.55	2416.12	1361.07	79.48	208.56
**Ethyl hexanoate**	0.01	Pineapple, fruit, flower	271.27	195.57	2409.69	2348.52	223.73	2249.32	490.87	360.08	2433.27	2370.17	3504.71	2269.71	233.88
**Ethyl octanoate**	0.00	Pineapple, pear, soapy	3796.78	2559.39	5880.15	6054.90	0.00	5609.07	2166.07	3820.71	9984.24	9295.55	11607.23	3897.05	2624.58
**Ethyl phenylacetate**	0.25	Honey	< 1	< 1	< 1	< 1	< 1	< 1	< 1	< 1	< 1	< 1	4.70	< 1	< 1
**Phenethyl acetate**	0.25	Rose, honey, tobacco	175.02	193.01	206.05	169.36	191.20	288.10	315.67	234.32	402.19	250.55	228.53	260.16	205.15
**Ethyl decanoate**	0.20	Fruit, fresh	111.83	77.45	77.00	76.41	45.26	94.45	43.91	97.62	131.72	122.87	121.86	38.05	44.84
**Ethyl laurate**	0.50	Flower and fruit aroma	8.84	10.22	11.12	9.21	5.44	12.52	10.07	9.36	23.73	17.85	23.11	3.12	5.53
**Linalool**	0.02	Flower, fruit, muscat	344.36	329.85	354.46	290.17	508.90	511.10	647.56	400.28	458.33	149.92	393.87	474.36	345.60
**4-Terpineol**	5.00	Pepper	2.46	2.49	2.75	2.39	1.69	2.01	2.41	3.06	0.00	2.73	2.79	3.49	2.41
**α-Terpineol**	9.18	Flower, lemon	1.94	2.16	2.34	1.86	1.79	2.32	2.54	2.43	2.77	2.04	2.34	2.70	1.61
**Citronellol**	0.70	Green, lemon	1.75	1.50	< 1	< 1	< 1	< 1	2.51	< 1	< 1	< 1	4.02	< 1	< 1
**Styrene**	0.16	Fruit, sweet	77.86	51.89	< 1	< 1	< 1	< 1	98.98	150.33	< 1	< 1	< 1	123.50	65.77
**D-Limonene**	0.20	Floral, green, tangerine	31.25	24.49	28.87	31.13	31.99	13.46	67.07	55.29	36.87	28.17	26.20	73.91	45.54
**Acetophenone**	0.01	tangerine	< 1	< 1	< 1	87.73	< 1	< 1	< 1	< 1	< 1	< 1	< 1	< 1	< 1
**Decanal**	0.01	Sweet, tangerine, flower	< 1	106.33	< 1	< 1	< 1	< 1	< 1	< 1	< 1	< 1	< 1	< 1	< 1
**Thymol**	0.05	Thyme	109.32	117.51	115.52	84.32	62.16	95.29	89.53	88.49	127.19	90.62	107.51	92.72	86.50
**Octanoic acid**	10.00	Rancid, harsh, cheese, fatty acid	< 1	< 1	< 1	< 1	< 1	1.17	< 1	< 1	< 1	< 1	< 1	< 1	< 1
**1,3-Xylene**	1.10	Strong aromatic smell	3.49	2.56	2.15	< 1	2.30	< 1	< 1	5.65	< 1	< 1	< 1	6.59	3.22

[a] The threshold of volatile aroma compounds referred to Van Gemert (2011); [b] The description of volatile aroma compounds referred to literatures [6, 20, 26, 32]; [c] The OAV of the compounds. Abbreviations: Sc, *S. cerevisiae*; Ce, *Candida ethanolica*; Hg, *Hanseniaspora guilliermondii*; Ht, *Hanseniaspora thailandica.*
